# Non-invasive measurement of biomolecular condensate interfacial tension and bending rigidity

**DOI:** 10.1016/j.crmeth.2025.101223

**Published:** 2025-11-11

**Authors:** Thomas A. Williamson, Jack O. Law, Thomas Stevenson, Fynn Wolf, Carl M. Jones, Endre S. Tønnessen, Sushma N. Grellscheid, Halim Kusumaatmaja

**Affiliations:** 1 Institute for Multiscale Thermofluids, School of Engineering, University of Edinburgh, Edinburgh, Scotland EH9 3FD, UK; 2 Computational Biology Unit, Department of Informatics, University of Bergen, 5006 Bergen, Norway; 3 Department of Biomedicine, University of Bergen, 5009 Bergen, Norway; 4 Department of Biosciences, University of Durham, Durham, DH1 3LE, UK

**Keywords:** biomolecular condensate, vesicle, flicker spectroscopy, interfacial tension, bending rigidity

## Abstract

Accurate measurement of biomolecular condensates’ mechanical properties is essential to understand their behavior within cells. We present *FlickerPrint*, an open-source Python package to determine the interfacial tension and bending rigidity of thousands of condensates using flicker spectroscopy by analyzing their shape fluctuations in confocal microscopy images. We detail the workflow and computational requirements of *FlickerPrint* to scale up these individual measurements to the population level. Examples of experiments in live cells and *in vitro* that are suitable for analysis with *FlickerPrint* are provided, as well as scenarios where the package cannot be used. Using these examples, we show that the results obtained are robust to changes in imaging setup, including frame rate. This implementation enables a step change in measurement capability for two key properties of biomolecular condensates: interfacial tension and bending rigidity. Moreover, the tools in *FlickerPrint* are also applicable for analyzing other soft, fluctuating bodies, demonstrated here using vesicles.

## Highlights

• We present a Python package to infer mechanical properties of condensates and vesicles
• The method is non-invasive and is applicable to bodies in live cells and *in vitro*
• Interfacial tension and bending rigidity are measured using high-throughput analysis
• We provide examples of suitable systems and considerations for imaging

## Motivation

Biomolecular condensates play fundamental roles in sub-cellular organization, and it is well known that the composition of condensates can affect their function. Measuring the condensates’ mechanical properties (for example, interfacial tension and bending rigidity) can aid the understanding of their biomolecular composition and cellular functions. However, measuring the properties of individual condensates under physiological conditions is very challenging and cumbersome to scale to the population level using traditional methods. To overcome these issues, we have developed a software package to run flicker spectroscopy analysis of condensates at scale, to determine their interfacial tension and bending rigidity. At the same time, the package can be harnessed to analyze other soft, fluctuating bodies such as lipid vesicles.

## Graphical abstract

**Figure:**
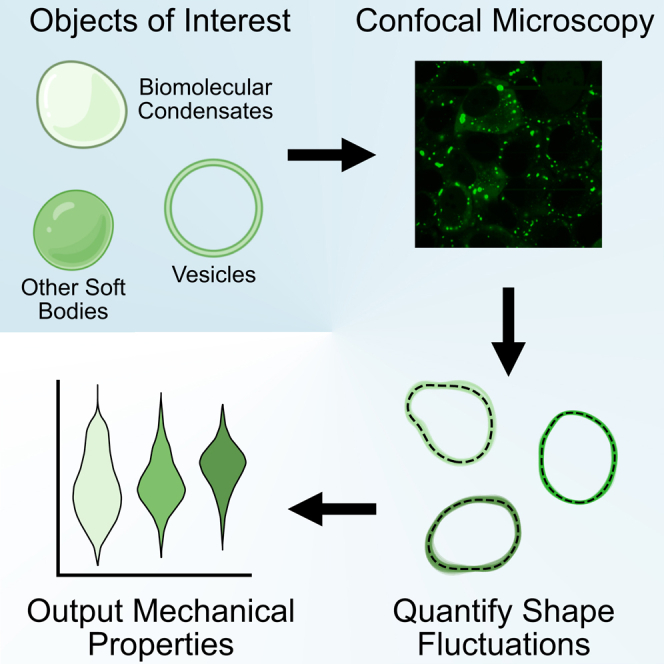

## Teaser

Williamson et al. present *FlickerPrint*, an open-source computational analysis tool for measuring the interfacial tension and bending rigidity of soft bodies from their shape fluctuations in time-series confocal microscopy videos. The package allows for thousands of biomolecular condensates, vesicles, and other soft bodies to be analyzed.

## Introduction

Biomolecular condensates are phase-separated, liquid-like droplets, which exist across a wide range of different environments, both *in vitro* as synthetic droplets and in cells as cellular sub-compartments such as stress granules, nucleoli, and p-bodies, where they regulate important functions from stress response to RNA transcription.^1^^,^^2^^,^^3^^,^^4^^,^^5^ One way to help to understand the behavior of condensates is by measuring their mechanical properties, a set of generalized parameters that govern the condensate’s mechanical response, from shape changes to droplet coalescence, wetting onto membranes, and production of biomolecules.^2^^,^^6^^,^^7^^,^^8^ In turn, the mechanical properties of a condensate are governed by its internal biomolecular interactions.^9^^,^^10^

Much work has focused on the bulk properties of condensates, showing that they are network fluids and that viscoelasticity plays an important role in governing their dynamics.^11^^,^^12^ However, the condensate interface has also been shown to play an important role in modulating chemical reactions, wetting, and the liquid-to-solid “aging” transition, which some condensates appear to undergo.^13^^,^^14^^,^^15^^,^^16^^,^^17^^,^^18^ The interfacial properties of condensates depend on the interactions between the molecules in the dense and dilute phases. Therefore, while *in vitro* techniques can provide highly accurate measurements of properties such as interfacial tension, these results may not translate directly to *in cellulo* assays.^19^^,^^20^^,^^21^ Non-invasive techniques allow for interfacial properties to be measured in live cells, pioneered with estimates of interfacial tension from the time taken for droplets to coalesce.^22^ However, since this technique relies on the observation of relatively rare coalescence events, it can be difficult to conduct at scale. More recent work has estimated the mechanical properties of coacervates by fitting a statistical model to their size distribution.^23^ While this can provide a useful population-level estimate of the average mechanical properties, it is not able to provide individual-level measurements to understand how parameters vary across an entire population.

To overcome these issues, we present *FlickerPrint*, a comprehensive, high-throughput Python package that implements the flicker spectroscopy method for measuring interfacial tension and bending rigidity (a measure of the elastic bending deformation of the interface) of condensates and other soft, fluctuating bodies such as vesicles.^6^ *FlickerPrint* non-invasively measures the mechanical properties of condensates or vesicles *in vitro* or in live cells using confocal microscopy images. These measurements are repeated at scale to produce parameter distributions for whole populations of the objects of interest, with individual-object resolution. The ability to collect measurements on a large scale using widely available microscopy setups will enable these assays to be carried out more routinely, so that the behavior of soft bodies such as condensates and vesicles can be better understood.

The flicker spectroscopy method determines the interfacial tension *σ* and bending rigidity *κ* by measuring the shape fluctuations of biomolecular condensates or vesicles in confocal microscopy images.^6^^,^^22^^,^^24^ By equating the Helfrich-type energy penalty of the shape fluctuations at the interface to the thermal energy of the object of interest, we can derive an analytic power spectrum of the amplitude V of the *q*^th^ fluctuation mode

(Equation 1) $$\left\langle {\left|V_{q}\right|}^{2}\right\rangle =\frac{k_{B}T}{\kappa }\sum_{l=q}^{l_{\max}}\frac{N_{lq}^{2}P_{lq}^{2}\left(0\right)}{\left(l+2\right)\left(l-1\right)\left[l\left(l+1\right)+\bar{\sigma }\right]}\text{,}$$

where *N*_*lq*_*P*_*lq*_(0) are the normalized Legendre polynomials evaluated at the condensate equator, $\bar{\sigma }=\frac{\sigma R^{2}}{\kappa }$, and *R* is the radius of the object.^25^^,^^26^ Taking *l*_*max*_ = 75 is typically sufficient for the sum to converge. Fitting this power spectrum to the experimentally determined fluctuation amplitudes allows the interfacial tension *σ* and bending rigidity *κ* to be determined as free parameters.^6^ The flicker spectroscopy technique was originally developed in the context of vesicles^27^^,^^28^ and red blood cells,^29^^,^^30^ and we recently demonstrated that it can be used to study biomolecular condensates.^6^

In this work, we outline the workflow used by *FlickerPrint* and describe the types of systems that are suited to analysis with the package. We then detail the parameters that can be measured using *FlickerPrint*, including interfacial tension, bending rigidity, and condensate shape, before describing the technical considerations when collecting microscopy videos for analysis. Finally, we detail how *FlickerPrint* can be installed and the computational requirements for running the package.

## Results

### Overview of the *FlickerPrint* package

*FlickerPrint* takes confocal microscopy images of condensates or other soft bodies (either in live cells or *in vitro*) and uses the flicker spectroscopy method that we have previously described to determine their interfacial tension and bending rigidity, among other properties.^6^ The package is available on all major platforms (Windows, Linux, and macOS) and can be run in either desktop or high-performance computing (HPC) environments.

**Figure 1:**
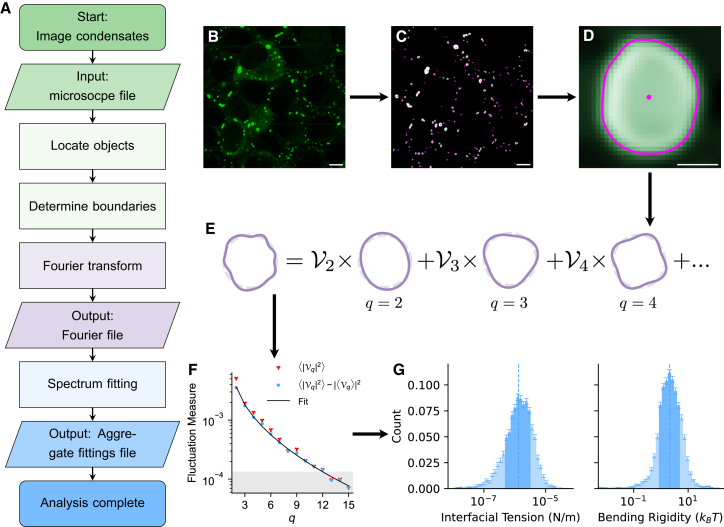
The workflow used by *FlickerPrint* (A) A flowchart outlining the key stages of the analysis. Colors indicate the stage of analysis: condensate detection and location (green), extraction of fluctuation Fourier components (purple), and power spectrum fitting (blue). (B–G) Illustrations of the key stages. (B) A raw micrograph input into *FlickerPrint* showing stress granules induced in U2OS cells using sodium arsenite. G3BP1 is fluorescently labeled. (C) The centers of condensates are located (magenta dots) using the difference of Gaussians method, and their approximate shape is determined using a flood fill (white regions). (D) The boundary of each condensate is determined as the maximal intensity gradient from the center of the condensate. (E) The magnitudes $V_{q}$ of the Fourier modes of the condensate boundary are determined for each condensate in each frame. (F) A power spectrum of the boundary fluctuations is produced and corrected for the base shape of the condensate. The interfacial tension and bending rigidity are the best-fit parameters of Equation 1 to the experimental spectrum. (G) The boundary determination, Fourier transform, and spectrum fitting steps are repeated for all condensates in the experiment to build up a population distribution of interfacial tension and bending rigidity. Error bars on the histograms are determined by propagating the standard error on the measured parameters for each condensate through the population distributions. Scale bars: 10 μm (B and C) and 1 μm (D). See also Figure S1.

A flowchart showing the basic workflow of *FlickerPrint* is shown in Figure 1A; the full control logic is shown in Figure S1. The workflow can be split into three main stages: (1) location of objects of interest, (2) extraction of the Fourier terms from the boundary fluctuations, and (3) fitting of the theoretical spectrum to determine interfacial tension and bending rigidity.

In the first stage, the objects are located and their boundary is determined. A difference of Gaussians (DoG) algorithm is used to detect condensates or vesicles as high-intensity regions relative to their local environment (Figure 1B).^31^ This allows for substantial variations in background intensity between cells to be accounted for, when applied in an *in cellulo* context. The size of the objects of interest to be found can be tuned by configuring the width of the Gaussian filters used, which can be useful to ensure that analysis of other cellular structures is avoided. The objects are tracked through frames in the video using a custom tracking algorithm (see STAR Methods), so that their shape fluctuations can be measured with time. Once the objects of interest have been located, a flood fill is used to determine their approximate extent and to draw a bounding box around them (Figure 1D). Next, the boundary of the object must be determined. In the microscopy frames, condensates appear to have a diffuse boundary. As such, the condensate boundary is taken as the maximal intensity gradient in the radial direction from the center of the condensate, using sub-pixel interpolation (see STAR Methods). For vesicles, the local maximum intensity of the image is used instead. This results in a function of the radius of the object at a polar angle *φ* about its center point (Figure 1D). For the images presented in this work, we find that the upper resolution limit for boundary detection is 1/15 of a pixel (see STAR Methods).

In the second stage, the boundary is normalized by the average radius of the object and broken into its constituent modes by taking a Fourier transform (Figure 1E) to yield the amplitude V of each Fourier mode *q*. Typically, modes 2 ≤ *q* ≤ 15 are used (*q* = 0 represents changing the size of the object and *q* = 1 represents translational movement, both of which are accounted for in the earlier stages of the analysis). For modes higher than *q* = 15, the amplitude of the mode is often close to the resolution limit of the boundary detection (Figure 1F), although the number of modes measured can be adjusted, if applicable.

Stages 1 and 2 are repeated for all of the input microscope videos. Intermediate files are produced, containing the locations, sizes, and boundary Fourier components of all objects in each frame of the videos.

The third main stage of the *FlickerPrint* workflow is fitting to the power spectrum. Simple Newtonian liquids have a spherical time-averaged base shape, so taking a Fourier transform of their boundary should only yield their thermal fluctuations. However, condensates and vesicles do not always have a spherical base shape. The shape of a condensate in a given frame is composed of two components: the time-averaged “base shape” of the condensate $\left\langle V_{q}\right\rangle$ and the instantaneous thermal fluctuations |*F*_*q*_| on top of this shape.

Therefore, the contribution of $\left\langle V_{q}\right\rangle$ is removed according to^32^

(Equation 2) $${\left|F_{q}\right|}^{2}=\left\langle {\left|V_{q}\right|}^{2}\right\rangle -{\left|\left\langle V_{q}\right\rangle \right|}^{2}\text{,}$$

to leave only the fluctuating contributions ${\left|F_{q}\right|}^{2}$. Equation 1 is fitted to the corrected experimental spectrum using a least-squares fit (Figure 1F) with a custom minimization function (see STAR Methods) to ensure that all orders contribute to the fitting. Interfacial tension *σ* and bending rigidity *κ* are the only free parameters in the fit.

A final “aggregate fittings” output file is produced, containing the interfacial tension and bending rigidity of all objects in the experiment, together with additional useful properties such as their mean radius and mean intensity. Population distributions such as those in Figure 1G can be produced using *FlickerPrint*’s built-in graphing tools or any other standard statistical analysis toolkit.

### Experiments suited to analysis with *FlickerPrint*

**Figure 2:**
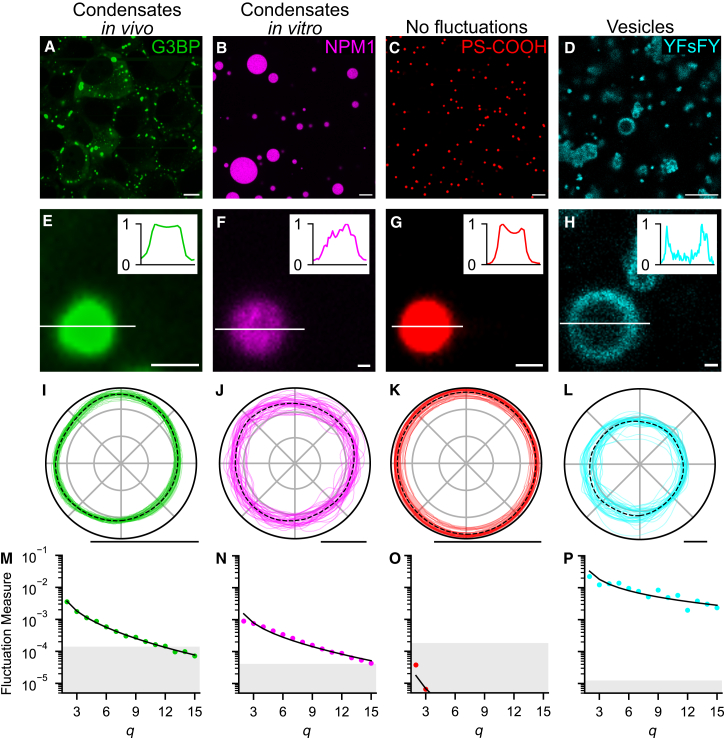
Examples of the suitability of different experimental setups for analysis with *FlickerPrint* (A–D) Confocal microscopy images of four different systems; stress granules in live U2OS cells (A), synthetic NPM1 condensates with a dextran crowding agent (B), fluorescent carboxyl-functionalized polystyrene particles (C), and coacervate-core vesicles (D). The fluorescently labeled species is given in each sub-figure. (E–H) Zoom-in of a single object of interest from (A)–(D). Insets give the intensity profile along the line shown. (I–L) Boundary fluctuations of the object shown in (E)–(H) with time. The black dotted line shows the time-averaged base shape of the condensate or vesicle. For clarity, only the first 40 fluctuations are shown. (M–P) The shape-corrected power spectrum for the fluctuations (dots) of the objects in (E)–(H) with the fit from the theoretical power spectrum shown as a black line. Fluctuation measure is normalized to the object’s mean radius. The shaded gray region denotes where the total fluctuation amplitude is too small to be resolved reliably (typically <0.067 pixels). (D), (H), (L), and (P) are reproduced with permission using data from Abbas et al.^24^ Scale bars: 10 μm (A–D) and 1 μm (E–L).

*FlickerPrint* takes microscopy videos as input, showing bright condensates or vesicles fluctuating against a dark background. Therefore, while our initial application of the method was to stress granules in live U2OS cells, the package can be used to characterize a variety of *in cellulo* and *in vitro* systems of both condensates and vesicles.^6^^,^^24^ Figure 2 shows four systems: a system of stress granules induced in U2OS cells using sodium arsenite (Figure 2A), an *in vitro* system of NPM1 droplets using a dextran crowding agent (Figure 2B), a system of solid fluorescent polystyrene particles (Figure 2C), and a system of coacervate-core vesicles, formed of peptides with a 2,2′-thiobis(ethylamine) spacer and two tyrosine-phenylalanine stickers (YFsFY) (Figure 2D).^24^ The condensates and polystyrene particles appear as bright, filled regions (Figures 2E–2G) whereas the vesicles appear as high-intensity outlines (Figure 2H); both intensity profiles can be used to determine the fluctuation spectrum of the objects of interest.

The fluctuations of each object are shown in Figures 2I–2L. Where the time-averaged base shape is non-spherical (Figures 2I and 2L), this contribution can be decoupled from the fluctuations using Equation 2.^32^ The fluctuations of stress granules, NPM1 condensates, and coacervate-core vesicles (Figures 2I, 2J, and 2L) are all large, compared with their radius. As such, the fluctuation spectra are almost entirely above the resolution guide (Figures 2M, 2N, and 2P). Therefore, these species are suitable for analysis with *FlickerPrint*. In contrast, the variations seen in Figure 2K are caused by movement of the bead into and out of the imaging plane, causing an apparent change in size but no change in shape. This does not impact the power spectrum. The lack of visible shape fluctuations means that the entire fluctuation spectrum of the polystyrene beads (Figure 2O) is below the resolution guide, so interfacial tension and bending rigidity cannot be determined using *FlickerPrint*, as expected for solid particles.

**Figure 3:**
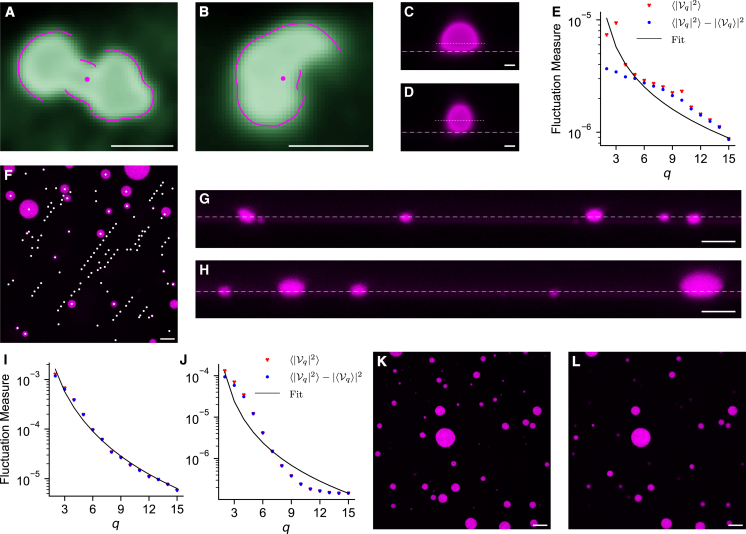
Ensuring that images are captured appropriately is essential for successful analysis using *FlickerPrint* (A and B) Examples of stress granules that have not passed the boundary filter as their boundary cannot be expressed as a continuous radial function (magenta line) about the center. (C) Side profile (*x*, *z*) confocal microscopy image of an *in vitro* NPM1 condensate with a dextran crowding agent that has wetted onto the surface of the imaging plate. The white dashed line indicates the location of the surface; the dotted line indicates the location of the equator (taken as the plane where the condensate radius is maximized). (D) Similar to (C) but where the wetting of the condensate onto the surface is minimal. (E) Example spectrum for a condensate where wetting onto the surface is significant. Note the poor quality of fit. (F) Image showing the effect on condensate tracking in FlickerPrint when condensates move too quickly. The effect is simulated by moving the microscope field of view in between frames. White dots show the location of “new” condensates found across all frames in the video. (G) Side profile (*x*, *z*) confocal microscopy image of an *in vitro* system of synthetic NPM1 (magenta) condensates with a dextran crowding agent, showing condensates of approximately the same size settled onto a slide. The white dashed line shows an example imaging plane passing close to the equator of the condensates. (H) Similar to (G) but for condensates that are not of an even size. In this case, it is not possible for the imaging plane to pass within ±0.34 radii of the equator of every condensate. (I) Example spectrum for an NPM1 condensate that has been imaged close to the equator. (J) Similar to (I) but for a condensate that has been imaged far from the equator, leading to a poor fit. (K) Top-down (*x*, *y*) confocal microscopy image of the *in vitro* NPM1 system, where the imaging plane has been selected to maximize the number of condensates imaged. (L) Similar to (K) but where the imaging plane has been moved up to capture the equatorial region of the larger condensates. Micrographs in positions similar to both (K) and (L) are required to produce suitable fluctuation spectra for both large and small condensates. Scale bars: 1 μm (A–D) and 10 μm (F–H, K, and L). See also Figure S2.

In addition to the objects having visible thermal fluctuations, they should also exist in thermal equilibrium and the object’s boundary must also be able to be described as a continuous radial function *D*(*φ*) of the polar angle about its center point, as shown in Figure 1D. This means that condensates that are undergoing fusion cannot be analyzed. Figures 3A and 3B show examples of stress granules in live cells that have been rejected because their boundary is not continuous. Often, where soft bodies are not in equilibrium or have external forces acting upon them, their shapes cannot be described as a continuous radial function (Figure 3A shows two stress granules fusing, for example). Condensates that have undergone aging to form solid-like aggregates also often fail this shape filter; however, since they are solid-like, they are unlikely to show thermal fluctuations above the resolution threshold of the microscope anyway.

The objects of interest must also exist in free space, meaning that they should not wet onto any cellular structures or their container. For example, Figure 3C illustrates an *in vitro* condensate that wets onto the bottom of its container, while Figure 3D illustrates a non-wetting condensate. The interactions between the condensate and the surface significantly impact the shape of the fluctuation spectrum (Figure 3E), meaning that it can no longer be modeled by Equation 1 and so must be discarded from the population-level statistics.

The final requirement of experiments to be analyzed using *FlickerPrint* is that the spatial position of the objects of interest should be relatively stable. *FlickerPrint* tracks the location of objects to account for small lateral movements in the XY imaging plane, up to a default displacement of 15 pixels per frame (see STAR Methods). If the objects move by more than 15 pixels per frame (as demonstrated in Figure 3F, where movement was simulated by moving the microscope field of view between frames), the objects may be lost by the tracking algorithm. If they are re-found far from their last known position in subsequent frames, they may be tracked as a new object, which may lead to the same object being analyzed multiple times, impacting the output parameter distributions.

It is also important to ensure that the *z* position of condensates remains stable, relative to the confocal microscopy imaging plane. In particular, Equation 1 assumes that condensates have been imaged at their equator, although the uncertainty in a condensate’s interfacial tension and bending rigidity is <20% when the imaging plane is within 0.34 radii of the condensate equator.^6^ Typically, this condition is met for condensates in live cells on a single microscope plate. Special care must also be taken when measuring the properties of *in vitro* condensates. It is preferable to allow *in vitro* condensates to settle onto the bottom of their container, to stabilize their *z* position, although the surface should be treated so that the condensates do not wet onto it. Where the surface cannot be treated to prevent wetting, floating condensates could be imaged, provided that the imaging is fast enough that their positions remain stable in *x*, *y*, and *z* throughout the video. If the objects of interest are of approximately uniform size, it is possible for the imaging plane to pass within 0.34 radii of the equator of all condensates (Figure 3G). However, when *in vitro* condensates are settled onto a surface but are not of approximately uniform size, choosing an imaging plane that maximizes the number of condensates in the frame (Figure 3H) will mean that larger condensates are imaged more than 0.34 radii from their equator. When condensates are imaged away from their equator, the shape of their fluctuation spectrum changes substantially (Figures 3I and 3J; Figure S2B), resulting in a significantly higher fitting error (0.073 for Figure 3I vs. 1.093 for Figure 3J) and leading to a poor estimate of the condensate’s mechanical properties. To combat this issue, images can be taken in two different planes (Figures 3K and 3L), which are suitable for imaging the equators of smaller condensates and larger condensates respectively. At the analysis stage, the condensates can be filtered according to size, to prevent double counting.

### Parameters that can be measured

**Figure 4:**
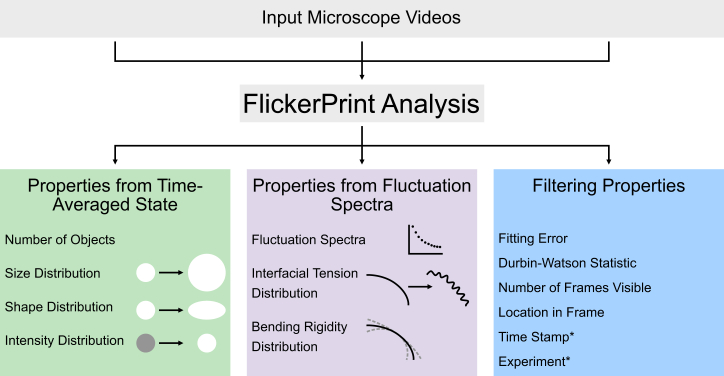
*FlickerPrint* allows the mechanical properties of condensates and vesicles to be determined at scale The properties can be grouped into three broad categories: those that are determined from the time-averaged state of the objects of interest, those that are determined from their thermal fluctuations, and additional properties that can be used for filtering population datasets. ∗Time stamp and experiment name are supplied from external metadata.

For experiments that are suitable, *FlickerPrint* can be leveraged to measure multiple properties of a population of condensates or vesicles. These properties are measured at the individual level and repeated at scale to provide parameter distributions at the population level. The analysis is parallelized across multiple microscope videos, where appropriate. The parameters that can be measured are summarized in Figure 4 and are broadly grouped into three main categories: properties that are determined by the time-averaged state of the object of interest, those that are determined by the fluctuation spectra of the object, and additional properties that can be used to filter the population dataset.

The first category of properties is determined from the time-averaged base shape of the condensate or vesicle and includes basic measurements such as the number of observed condensates and their size and intensity distributions. These are regularly quantified in the literature and can be measured by existing packages.^33^ Together, they can provide valuable information about the propensity of a system to phase separate.^34^^,^^35^ FlickerPrint allows this analysis to be easily parallelized across multiple microscope videos from the same assay. However, *FlickerPrint’s* capabilities go beyond these standard measurements; less-readily characterized is the shape distribution of populations of condensates and vesicles. Condensates are understood to take on irregular shapes when they age to form solid-like aggregates or when they are deformed by other cellular components such as fibrils.^14^^,^^36^^,^^37^ However, stress granules also appear to take on non-spherical base shapes, which may be evidence of their viscoelastic behavior and structured interface.^6^ In the context of vesicles, the base shape may provide information on the membrane area to volume ratio of the system.^38^

The second set of properties are those determined from the fluctuation spectrum of the objects of interest. These include the raw fluctuation spectra themselves, as well as the interfacial tension and bending rigidity derived from the spectra. Flicker spectroscopy has previously been used to successfully measure the properties of individual vesicles and red blood cells.^27^^,^^28^^,^^29^^,^^30^^,^^39^ However, to the authors’ knowledge, *FlickerPrint* is the only software package that can measure the interfacial tension and bending rigidity of individual biomolecular condensates and vesicles at scale to allow for population-level analysis. Therefore, both the mean properties and shape of the property distributions can be determined and compared, to understand how properties interact to influence condensate behavior. Recent estimates of mean interfacial tension from the size distribution of CPEB4Δ4_NTD_ coacervates give values of 1–1.5 μN/m, and measurements of bending rigidity for fused in sarcoma (FUS) protein and *α*-synuclein clusters give values of 0.7–1.3*k*_*B*_*T*.^23^^,^^40^ These are in broad agreement with the values that we have previously found for stress granules in U2OS cells.^6^

The final set of properties are those that are produced as a result of the data collection and analysis and can be used for filtering when visualizing population-level parameter distributions. These include the fitting error and Durbin-Watson statistic (a measure of the autocorrelation between residuals associated with fitting Equation 1 to the fluctuation spectrum). Examples of spectra with good and poor fitting error and Durbin-Watson statistic are shown in Figure S3. The number of frames that an object of interest is visible for can also be used as a filter to ensure that only objects with enough shape fluctuations to be properly time averaged are included in the parameter distributions. In addition, the image timestamp, a user-defined experiment name (both supplied externally), and the location of objects within the frame can be used to aid in the comparison of parameters measured in different assays. A full list of parameters that can be measured is available in the *FlickerPrint* documentation.^41^

### Imaging considerations

*FlickerPrint* is compliant with the Euro-BioImaging FAIR standard and can accept image files in one of the following formats: *.ims*, *.lif*, *.ome.tif[f]*, or *.tif[f]*.^42^ It may be possible to use other Bioformat-compliant file types, but these are untested.^43^ It is recommended that unsupported file types be converted to *.tif* or *.ome.tif* files prior to analysis. In addition, some of the above file types may be missing important metadata and must be handled carefully; *.tif* files do not contain the pixel size in their metadata, so this must be recorded manually from the microscope and provided explicitly to *FlickerPrint* via the analysis configuration file.

The measured interfacial tension and bending rigidity of a single population of condensates can span multiple orders of magnitude.^6^ Therefore, a large sample size is required to sufficiently map out the parameter distribution at the population level. For a single assay, it is generally best to generate at least 10 videos by moving the microscope field of view around the sample, focusing on areas with a high density of condensates. A final count of above 1,000 analyzed condensates may require up to 50 input videos, depending on the condensate density and the fraction of those condensates that pass the filtering steps. A sample image is available in Williamson et al.^44^

**Figure 5:**
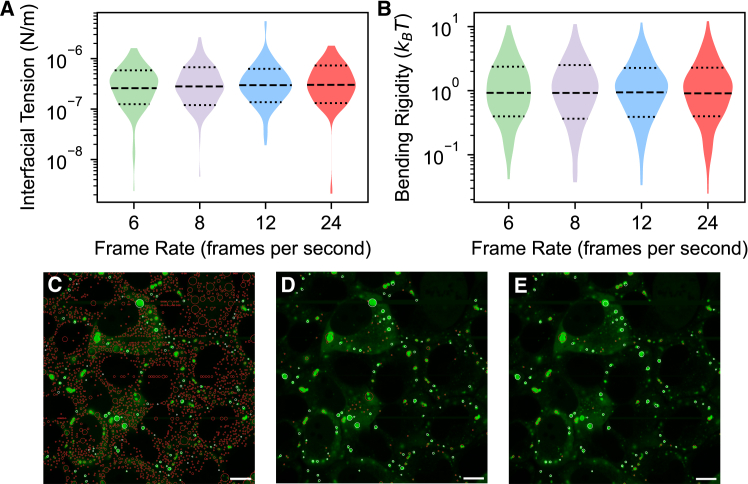
Ensuring that microscope videos are captured and imaging parameters are configured effectively leads to the highest chance of successful use of *FlickerPrint* (A and B) Violin plots showing the distribution of interfacial tension and bending rigidity for stress granules induced using sodium arsenite in U2OS cells, analyzed at effective frame rates of 6, 8, 12, and 24 frames per second. Two videos were taken, each at 24 frames per second, and every *n*^th^ frame was analyzed in order to produce the effective frame rates shown. (C–E) The effect of imaging parameters on the number of found condensates; microscope images of stress granules induced in U2OS cells, with the location of condensates found by *FlickerPrint*. Red circles show condensates that go on to be rejected by the boundary detection algorithm in this frame; white outlines show condensates that are accepted. The images show the effect of changing the minimum intensity parameter; (C)–(E) show intensity thresholds that are too low, optimal, and too high, respectively. Scale bars: 10 μm (C–E).

In addition to the total number of objects of interest that are analyzed, it is also important to try to maximize the total number of frames each object is visible for; each object should ideally be visible for at least 200 frames, with ∼1,000 frames being optimal. This ensures that the magnitudes of the fluctuations can be properly averaged, as required by Equation 1. The frame rate used to capture the micrographs should be high enough to ensure that the imaging timescale is much smaller than the timescale of condensate aging or any other changes in the mechanical properties (typically corresponding to a frame rate of 20–25 frames per second). However, provided this condition is met, the frame rate used does not substantially affect the measured properties of the condensates, as demonstrated in Figures 5A and 5B and Table S1; the mean interfacial tension and bending rigidity deviate by 0.043 μN/m and 0.03*k*_*B*_*T* (corresponding to <0.3 and <0.06 geometric SDs of the population distributions), respectively, when comparing condensates that are found and pass the necessary filters in all four analyses. When stress granules that were not found across all four analyses are included, the mean interfacial tension and bending rigidity deviate by 0.12 μN/m and 0.19*k*_*B*_*T* (corresponding to <0.5 and <0.3 geometric SDs of the population distributions) respectively (Table S2), indicating that the differences can largely be attributed to an increased number of condensates being found and successfully tracked at higher frame rates.

To account for differences in contrast, size, and intensity of objects of interest in the videos, the directory of each experiment contains a configuration file that allows key imaging parameters to be adjusted. In particular, four parameters control the approximate maximum and minimum size of the detected objects, their minimum intensity, and the flood fill used to determine their approximate extent. Since these parameters are used to locate objects of interest in each frame and do not directly impact the determination of the object boundaries, the analysis is relatively robust to their variation. When parameters are within their optimal range (the approximate range of values that maximize the number of objects found while minimizing the number that do not go on to pass the boundary continuity requirement), interfacial tension and bending rigidity vary by < 0.42 and <0.52 geometric SDs, respectively (Figure S4). These variations are most likely due to differing condensates detected in each frame, as a result of the imaging parameters used, rather than differences to the determined boundary. A full discussion of the effect of imaging parameters on the returned property distributions is provided in STAR Methods.

Once objects of interest have been located, their boundary is determined independently. Boundary detection is controlled by a single configurable parameter, which acts to smooth the input images to account for microscope noise (STAR Methods). Typically, this parameter should be kept as close to 1.0 as possible, while minimizing the number of objects where the detected boundary is not continuous.

Nevertheless, it is important that optimal imaging parameters are selected to maximize the number of condensates that can be analyzed, while minimizing the number of false detections that increase computational expense (Figures 5C–5E). To assist with selecting the optimal parameters for a given set of images, *FlickerPrint* contains a Bayesian optimization tool. This tool implements a lexicographic optimization to maximize the number of condensates found, while minimizing the number that do not go on to pass the later boundary filters (see STAR Methods).^45^^,^^46^

### Running *FlickerPrint*

*FlickerPrint* is available as a Python package for all major operating systems (macOS, Windows, Linux) and can be installed directly through pip; the package is open-source and is also available on GitHub.^47^ *FlickerPrint* requires Python 3.9-3.11 and Java 11.0.23 or later. Installation instructions and further guidance can be found in the documentation.^41^ A sample image is also available.^44^

*FlickerPrint* analysis can be scaled with core count, up to a maximum of one core per microscope image; the main analysis is executed through the command line using a single command.

**Figure 6:**
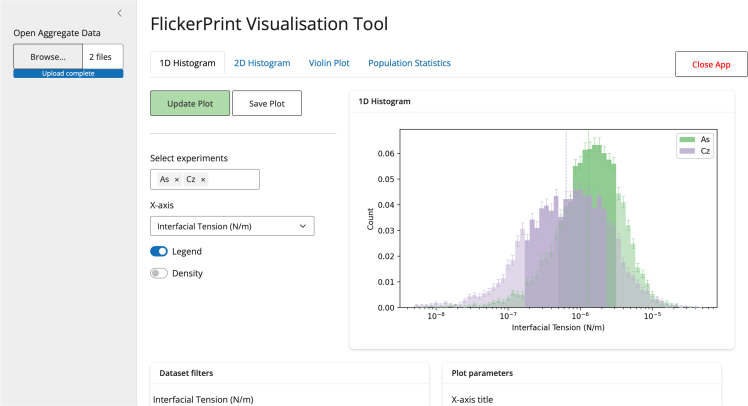
*FlickerPrint*’s auxiliary tools improve accessibility for users who may not have a strong computational background A screenshot of the graphical user interface application for producing plots of the parameter distributions from data output by *FlickerPrint*. Data used in these plots are reproduced with permission from Law et al.^6^

Data are analyzed as an experiment, composed of one or more microscope videos of the same biological system, and defined by a single configuration file that contains the settings required for conducting the analysis. The experiment directory is also the location where intermediate and final output files are written to. All output files are saved in the HDF5 format, with data saved as standard c-type values, allowing them to be read by any standard statistical analysis software.^48^ However, an additional graphical user interface tool is also provided (Figure 6). This allows for the production of 1D and 2D histograms so that the parameter distributions can be visualized and potential correlations between parameters can be found (see Figure 4 for examples of properties that can be measured using *FlickerPrint*). In addition, distributions from multiple experiments can be analyzed simultaneously using violin plots, allowing for the comparison of the effect of different treatments or biomolecular compositions on the mechanical properties of the object.

## Discussion

In this work, we have presented *FlickerPrint*, an open-source Python package for undertaking flicker spectroscopy analysis of soft bodies at scale. We have demonstrated that this package can be used to measure the properties of biomolecular condensates and coacervate-core vesicles, both in live cells and *in vitro*. We have also noted the requirements of experimental setups and microscope videos in order for them to be successfully used with *FlickerPrint*.

Principally, *FlickerPrint* is intended to measure interfacial tension and bending rigidity from the shape fluctuations of the bodies. Many works have suggested that biomolecular condensates are viscoelastic droplets. Measuring both interfacial tension and bending rigidity using *FlickerPrint* supports the idea that biomolecular condensates have structured interfaces for example, as described by the core-shell and protein conformation models.^12^^,^^23^^,^^40^^,^^49^ Since *FlickerPrint* requires the objects of interest display thermal fluctuations above the resolution threshold, it is best-suited for condensates with more liquid-like morphologies. It may be possible to extend the method in the future to estimate properties such as viscosity, though this introduces additional requirements such as a dependence on the frame rate of the video.^50^ However, aside from interfacial tension and bending rigidity, the time-averaged state of the objects of interest can also be used to extract information on their size, number, and shape, including for condensates with more solid-like morphologies, where interfacial tension and bending rigidity measurements are not possible. All of these properties can be measured as distributions at the population level, with individual-object resolution.

*FlickerPrint*, as a package for conducting flicker spectroscopy analysis, aims to make large-scale surveys of the mechanical properties of condensates and vesicles more accessible. The scalable architecture used by *FlickerPrint* allows the package to be used in both desktop computing environments for testing and small-scale experiments as well as HPC environments for large-scale assays. The auxiliary tools for selecting optimal imaging parameters and analyzing population-level data included in the package further improve its ease of use.

The confocal microscopy setup required for image collection for *FlickerPrint* is typically much more readily available in experimental biology laboratories than the setups required for du Noüy ring and similar experiments.^19^^,^^20^ We anticipate that where changes are required to produce images of suitable quality for *FlickerPrint* (for example, by adjusting the imaging plane position), these should be easy to implement and should not require much more work than for collecting images for other purposes.

Measuring the coalescence time of condensates is another popular, non-invasive technique for estimating their interfacial tension.^22^^,^^51^ While coalescence events themselves are typically quite fast, they are also relatively rare, meaning that these measurements provide a useful estimate of interfacial tension on long timescales for systems that do not evolve quickly in time. In contrast, the time scales required for image collection in *FlickerPrint* are of the order 1 min. This may allow the properties of a population of condensates to be tracked with time, for example, during aging or other dynamic processes, where the process timescale is much greater than the imaging time. Dynamic light scattering experiments can also be used to measure the size distribution of condensates, from which an estimate of interfacial tension and bending rigidity at the population level can be determined by fitting a statistical model that accounts for differing protein confirmations at the interface and in the bulk of the condensate.^23^ While this method can be used at scale, it can only provide an estimate of the mechanical properties of the system at the population level.

We believe that flicker spectroscopy acts as a complementary method to those described above for measuring the interfacial properties of individual biomolecular condensates, which can be scaled up to provide information about the mean and shape of the property distributions at the population level. *FlickerPrint*, as a package for completing the flicker spectroscopy analysis, makes the technique more accessible by implementing the analysis pipeline and handling the scaling required to measure the properties of populations of biomolecular condensates or vesicles.

### Limitations of the study

Throughout this work, we have outlined scenarios where it is not possible to use *FlickerPrint* (or the flicker spectroscopy method more generally) to determine the properties of a population of condensates. The first principle limitation is that condensates must not have solid morphologies, to ensure that their shape fluctuations can be measured (Figure 2O). We have previously shown that as stress granules age, fewer granules are able to pass the necessary filtering steps to complete the flicker spectroscopy analysis.^6^ The second principle limitation is that condensates must exist in free space (so cannot be wetted onto containment vessels or cellular structures, for example) as these would provide additional energy contributions that would need to be accounted for in the fluctuation spectrum. However, even where it is not possible to accurately determine the interfacial tension and bending rigidity, *FlickerPrint* can still provide basic information on the size, circularity, and fluorescence intensity distributions of the species being measured.

## Resource availability

### Lead contact

Requests for further information and resources should be directed to and will be fulfilled by the lead contact, Halim Kusumaatmaja (halim.kusumaatmaja@ed.ac.uk).

### Materials availability

This study did not generate new unique reagents.

### Data and code availability

• A sample video for use with the FlickerPrint package is available on the BioImage Archive.^44^ Note that for portability reasons, this video is shorter (133 frames) than the recommended length (1,000 frames). All other raw imaging data generated for this study are available from the lead contact upon request.
• Source code for *FlickerPrint* is available on GitHub (https://github.com/FlickerPrint/FlickerPrint or https://doi.org/10.5281/zenodo.17077694) and can be installed directly using PyPI, the Python Package Index (https://pypi.org/project/flickerprint/). Documentation for *FlickerPrint* can be found at https://flickerprint.github.io/FlickerPrint/.
• Any additional information required to re-analyze the data reported in this paper is available from the lead contact upon request.

## Acknowledgments

We thank E. Spruijt, Radboud University, for reagents and helpful discussions; N. Kedersha, Harvard University (retired), for reagents; N.A. Yewdall, University of Canterbury, for support regarding the *in vitro* condensates; and M. Turner, University of Warwick and H. Dale at the Molecular Imaging Center, University of Bergen, for insightful discussions. We also thank ARC at Durham University for the use of the Hamilton8 HPC service and EPCC at the University of Edinburgh for use of the Cirrus HPC service, which have both supported this work. The graphical abstract has been created by T.A.W. using figures from BioRender (https://BioRender.com/prys28t). S.N.G., H.K., T.A.W., T.S., and C.M.J. acknowledge funding from the 10.13039/501100005416Research Council of Norway (grant no. 335901). H.K. acknowledges funding from 10.13039/501100000266UKRI Engineering and Physical Sciences Research Council (grant no. EP/V034154/2). S.N.G. acknowledges support from the 10.13039/100016190Trond Mohn stiftelse (no. BFS2017TMT01) and 10.13039/501100000288The Royal Society, UK. S.N.G. and F.W. acknowledge funding and support from the 10.13039/501100005036University of Bergen.

## Author contributions

J.O.L., T.A.W., C.M.J., F.W., and E.S.T. wrote the software. T.S. and T.A.W. performed the experiments. T.A.W. and F.W. performed the *FlickerPrint* analysis. H.K. and S.N.G. supervised the project. T.A.W., J.O.L., T.S., and F.W. prepared the manuscript. H.K. and S.N.G. edited the manuscript. All authors were involved in discussions to develop the software, experiments, and final manuscript.

## Declaration of interests

The authors declare that they have no competing interests.

## STAR★Methods

### Key resources table

**Table:** 

REAGENT or RESOURCE	SOURCE	IDENTIFIER
**Chemicals, peptides, and recombinant proteins**		

Purified NPM1	Yewdall et al.^52^	PMCID: PMC9674983; Yewdall et al.^53^
PS-COOH	Spruijt et al.^54^	DOI: https://doi.org/10.1039/C1SM05881A

**Deposited data**		

Sample Image	Williamson et al.^44^ https://www.ebi.ac.uk/biostudies/BioImages/studies/S-BIAD1763	DOI: https://doi.org/10.6019/S-BIAD1763

**Experimental models: Cell lines**		

U2OS ΔΔG3BP1/2 + GFP-G3BP1	Kedersha et al.^54^	Kedersha et al.^54^ PMCID: PMC4810302.

**Recombinant DNA**		

pET28a(+)-NPM1-WT	Yewdall et al.^52^	PMCID: PMC9674983; Yewdall et al.^53^

**Software and algorithms**		

FlickerPrint (Python Package)	This paper	https://github.com/FlickerPrint/FlickerPrint DOI: https://doi.org/10.5281/zenodo.17077694

**Other**		

Microscope Hardware (XY videos): Andor Dragonfly 505 spinning disk confocal microscope. • 100×1.49 Numerical Aperture CFI SR HPApo total oil immersion objective • iXon 888 Camera • Zyla 4.2 PLUS sCMOS camera		N/A
Microscope Hardware (XZ micrographs): Leica TCS SP8 confocal microscope • 100×1.4 NA HC PL APO STED WHITE oil immersion objective		N/A

### Experimental model and study participant details

#### Condensates in live cells

U2OS ΔΔ G3BP1/2 stably expressing GFP-G3BP1 were a gift from N Kedersha.^54^ U2OS cells were cultivated in Dulbecco’s modified Eagle’s medium (DMEM) (Sigma-Aldrich, no. D5671) containing 10% fetal bovine serum, 100 μg/mL penicillin/streptomycin at 37°C, 5% CO2. For imaging, cells were passaged and cultivated in 35 mm glass-bottom dishes (Ibidi, cat. no. 81158) or 18-well glass-bottom slides (Ibidi, cat. no. 81817) and stress granule condensates were induced via incubation in 200 *μ*M sodium arsenite.

### Method details

#### *In vitro* NPM1 condensates and polystyrene particles

Reconstituted NPM1 condensates were formed in a buffer consisting of 20 mM Tris-HCl [pH 7.2], 250 mM potassium glutamate supplemented with 5% Dextran. The final concentration of NPM1 protein in each experiment was 30 μM. Purified NPM1 and the NPM1 bacterial expression construct were provided by E Sprujit and was purified, labeled, and prepared as previously described.^52^ Fluorescent (FITC) carboxyl-functionalised polystyrene particles were provided by E Sprujit and diluted in MQ water for imaging.^53^ Samples for *FlickerPrint* were imaged in functionalised 18-well glass-bottom imaging slides (Ibidi, cat. no. 81817), which were cleaned using a plasma cleaner and incubated overnight with 0.1 mg/mL PLL(2)-g[3.5]-PEG(2) (SuSoS, Dübendorf, Switzerland) dissolved in 10 mM HEPES (pH 8.0).

#### Microscopy

Videos for *FlickerPrint* analysis in the XY plane for stress granule condensates in live cells, *in vitro* NPM1 condensates, and polystyrene particles were acquired using an Andor Dragonfly 505 spinning disk confocal system with a 100 ×1.49 numerical aperture (NA) CFI SR HPApo total oil immersion objective using an iXon 888 or Zyla 4.2 PLUS sCMOS camera. XZ confocal micrographs of NPM1 condensates in Figures 3C and 3D were acquired using the same setup as for the *FlickerPrint*-quality images. A z stack with one frame every 0.01 *μm* was taken and converted into XZ images using ImageJ.^33^ XZ confocal micrographs of NPM1 condensates in Figures 3G and 3H were acquired using a Leica TCS SP8 confocal microscope with a 100×1.4 NA HC PL APO STED WHITE oil immersion objective. Distortion in the XZ micrographs was manually corrected via comparison to polystyrene particles of known size.

#### Object tracking

As discussed in the main text, in each frame of the input microscope file, objects of interest (condensates or vesicles) are detected using the Difference of Gaussians method.^31^ The next step is to match objects between frames, so that per-object averages can be calculated at later steps. This tracking process poses some unique challenges in the case of *FlickerPrint*. Here, we outline the bespoke tracking algorithm used by *FlickerPrint*.

The greatest challenge for designing the tracking algorithm is that the Difference of Gaussians detector is not fully reliable, which means that an object may be missed for a few frames, from time to time. Therefore judgments must be made about whether a newly detected object is genuinely newly-appeared, or whether it is a previously detected object that has been dropped for a few frames. As a result, the tracking does not only match to objects that occurred on the previous frame, but retains a memory of the last known position of each object, that endures for 10 frames.

Conversely, as the objects should all lie on a plane, and we do not expect them to move very quickly (and ideally not at all), we are absolved from the need to deal with crossing trajectories or ballistic motion. Therefore, we have designed a tracking algorithm that is based on object positions only; ignoring velocity or shape-matching.

The *FlickerPrint* tracking algorithm is described below.

(1) On the first frame, there is no need for tracking. Each object detected is assigned a unique integer ID, starting from 0 and counting up. The position and ID of each object is stored in the tracker’s memory for comparison with future frames.
(2) On subsequent frames, we begin by looking for objects that appear both in the tracker’s memory and the current frame. An object in the current frame is identified as an object in the tracker’s memory if their centers are within 15 pixels of each other, and they are closer than any other object. The ID of the object in the current frame is set to the ID stored in the tracker’s memory, and the position stored in the tracker’s memory is updated to the latest position. If the object is one that was previously missing, then its countdown is stopped and deleted (see step 4.).
(3) This leaves two classes of unhandled object: objects that appear in the current frame but not in the tracker’s memory, and objects that appear in the tracker’s memory but not the current frame. The first class are newly appeared objects. They are assigned a currently-unused ID and added in to the tracker’s memory.
(4) The other class are the missing objects. If an object is newly missing (i.e., it appeared in the previous frame), a countdown is started from 10, but the object remains in the tracker’s memory at its last known position. If the object already has a countdown (i.e., it has been missing for more than one frame), then the countdown is decremented by one. If the countdown reaches zero the object is deleted from the memory permanently. Therefore, if an object subsequently appears at that position, it will be counted as a new object.

The memory value of 10 frames is chosen so that an object that is missed by the detector for a few frames is usually re-identified, but that it is very rare that a granule will drift onto the position of a previously-vanished object and be miss-identified as the same object.

The objects are tracked up to a default distance of 15 pixels from their last known location. Typically, diffusion of the objects of interest is low, so plays little role in the movement of condensates between frames. The default distance of 15 pixels was chosen to be sufficient to account for typical rates of advection of soft bodies seen during development. Advection of the bodies is usually highest *in vitro* but can be reduced by allowing them to settle (but not wet) onto the surface of their container.

The value of this threshold can be changed in the experiment configuration file, though it is advised not to change this value unless there are issues tracking the objects of interest between frames. The effect of altering this threshold is shown in Figure S5 for both the system of stress granules shown in Figure 2 and the system of *in vitro* condensates shown in Figure 2 of the main text. In both cases, the number of condensates found remains similar when the tracking threshold is above 12 pixels.

#### Boundary detection

In order to estimate the boundary of the objects of interest, we must first determine the extent of the object in the frame. Using the center point found by the difference of Gaussians algorithm, we use a simple flood fill to estimate the extent of the object, and use this as a guide to cut out a rectangular region containing just that object. The extent of the flood fill can be set in the configuration file.

We then smooth the image by applying a Gaussian blur with a width set in the configuration file. This reduces internal gradients caused my noise in the microscope file. The procedures described above are the same for both vesicles and condensates. However, at this point condensates require an additional processing step. We take the directional gradient $g=\nabla I\cdot \hat{r}$, where *I* is the image and $\hat{r}$ is the radial unit vector field from the condensate center. We estimate ∇*I* using a fourth order kernel, given by $k_{4\text{th}}=\frac{1}{12}\left(\begin{array}{ccccc} 1 & -8 & 0 & 8 & -1 \end{array}\right)$. This kernel is applied to the input image as a stencil, centered at each pixel, and multiplied element-wise.^55^ As we expect *g* to be highest at the edge of the condensate, the following steps are once again the same for vesicles and condensates.

To find the outline of the object, we emit 400 rays radially from the center point of the object. We sample the processed image intensity at equally spaced points along these rays at a density 15 per pixel-width. We use a fourth-order interpolation to obtain values resolved at a sub-pixel level. For each ray, we take the sampled point with the highest intensity value. This leaves with 400 points on the frame that describe the outline of the object.

#### Justification of the resolution of boundary detection

As described in the main text, for objects which appear as solid regions of high intensity (rather than high intensity outlines) such as condensates (main text Figures 2E and 2F), the boundary is taken as the contour of maximum intensity gradient, measured radially from the center of the object. The condensate boundary can be determined to a resolution of 1/15 of a pixel. Here, we justify this claim.

The normalised 1-Dimensional intensity profile of the boundary of a condensate can be represented by

(Equation 3) $$I\left(x\right)=\frac{1}{2}-\frac{1}{2}\tanh\left(\frac{x-x_{0}}{\zeta }\right)$$

where *x* is an integer representing the position of a pixel, *x*_0_ is the ‘true’ position of the boundary and *ζ* is the interface width; typically *ζ* = 0.8. An image with 8-bit colour-depth contains 255 intensity levels per pixel. Requiring a difference of 10 levels in order to determine the boundary position ensures that the intensity gradient is sufficiently steep to avoid mis-detections and provides allowances for when the contrast in an image is not optimal. Therefore, the difference in the intensity of a pixel at *x* when the boundary is at locations *x* and *x*+*α* is

(Equation 4) $$\frac{1}{2}\tanh\left(\frac{x-x}{\zeta }\right)-\frac{1}{2}\tanh\left(\frac{x-\left(x+\alpha \right)}{\zeta }\right)=\frac{n_{levels}}{255}\text{.}$$

Simplifying down gives

(Equation 5) $$\tanh\left(\frac{\alpha }{\zeta }\right)=2\frac{n_{levels}}{255}\text{.}$$

Substituting in values gives *α* = 1/15.9. Thus, we determine the condensate boundary to a resolution of 1/15 of a pixel.

#### Spectrum fitting

The final step in calculating the interfacial tension and bending rigidity of a condensate or vesicle is fitting the experimentally measured fluctuation spectrum (main text, Figure 1F) to Equation 1 of the main text. Firstly, we must define an error function *ε*, which allows us to compare the quality of fits. We previously showed that the best error function for this problem is

(Equation 6) $$\varepsilon =\sum_{q}\left|{\left(\log_{10}\left[\frac{{\left|F_{q,\text{theo}}\right|}^{2}}{{\left|F_{q,\exp\;}\right|}^{2}}\right]\right)}^{2}\right|\text{,}$$

where |*F*_*q*,theo_| is the theoretical fluctuation spectrum and |*F*_*q*,*exp*_| is the experimental spectrum.^6^ This value is minimised to find the best fit for *σ* and *κ*.

Figure S6 shows a representative fitting surface for a stress granule, across several orders of magnitude. It can be seen that there is only one minimum, which means that down-hill fitting methods are sufficient to find the global minimum. Therefore, fitting is carried out as follows.

(1) We generate an initial guess by sampling $\bar{\sigma }$ and *κ* across 14 orders of magnitude on a logarithmic scale. The pair that gives the lowest *ε* is used as a starting point for the next step.
(2) We then use the least_squares function from the scipy.optimize python module to find the true minimum of *ε*.^56^

#### Robustness of *FlickerPrint* analysis to changes in imaging parameters

As discussed in the main text, *FlickerPrint* contains five parameters which can be adjusted to account for differences in imaging setup. These are the minimum intensity (relative to the frame maximum intensity) which an object must have in order to be detected, its approximate maximum and minimum size, the threshold intensity of the flood fill and a smoothing parameter which is used to account for microscope noise. Choosing appropriate parameters ensures that the maximum number of objects of interest are found in each frame, whilst minimising the number of false positive detections. However, here we show that the results of *FlickerPrint* analysis are relatively robust to changes in these parameters.

Figure S4 shows the effect of systematically varying each of the imaging parameters for an image of stress granules in U2OS cells. In each case, the final number of condensates analyzed after the parameter distributions have been filtered, and the returned distributions of interfacial tension, bending rigidity and mean condensate radius are plotted. When allowing the imaging parameters to be varied across a wide range of values, interfacial tension, bending rigidity and mean radius vary by up to 1.3 *μN*/*m*, 12 *k*_*B*_*T* and 0.75 *μm* respectively. To allow for direct comparison, it can be helpful to quote the variation in the population distributions in terms of their geometric standard deviation (for interfacial tension and bending rigidity) and standard deviation (for mean radius). When quoted as a fraction of the standard deviation of the two parameter distributions in least agreement, interfacial tension, bending rigidity and mean radius vary by < 1.3 SD, <4.1 SD and <15.3 SD respectively (Figure S4). However, when the parameters are within their optimal range (defined as the approximate range of parameters which maximise the number of detected objects, whilst minimising the number of objects which fail the later filtering steps), the variation in the interfacial tension, bending rigidity and mean radius distributions for all condensates is reduced to <0.52 SD, <0.42 SD and <0.93 SD respectively.

Comparing the final population distributions can be useful to gauge the variation in population distributions caused by changes in the imaging parameters. However, since changing the imaging parameters affects the number of condensates which are found, comparing the populations of all condensates does not provide much information on how the properties of individual condensates are affected. Therefore, Figures S4A–S4D also shows the returned parameter distributions when only condensates which are found and pass the filters in at least 75% of analyses are included. A cut-off of 75% ensures that the distributions contain broadly the same condensates, whilst allowing flexibility to ensure that the population distributions are large enough for statistical analysis. In this case, the parameter distributions for interfacial tension, bending rigidity and mean radius deviate by < 0.89 SD, <1.1 SD and <1.9 SD respectively across the full range of imaging parameters, and <0.28 SD, <0.45 SD and <0.12 SD respectively when only optimal parameters are considered. These variations in the returned distributions are much less than for the case where all condensates are considered. This supports the idea that the variations are largely due to different condensates being found when the imaging parameters are changed, since these parameters control where objects are found in the image, not the position of their boundary.

Once objects have been found, their boundary is determined. This process is configured by a single parameter which smooths the image to account for microscope noise which would cause discontinuities in the object boundary. Similar analysis to that conducted for the parameters responsible for object location has been conducted for the smoothing parameter and is shown in Figure S4E. In this case, the typical variation in the returned parameter distributions across the full range of smoothing values is small compared with the imaging parameters used for locating objects (<0.61 SD, <0.31 SD and <2.3 SD for interfacial tension, bending rigidity and mean radius). However, the distributions from condensates found in at least 75% of analyses do not see the same reduction in variation as for the other imaging parameters (<0.41 SD, <0.76 SD and <0.87 SD for interfacial tension, bending rigidity and mean radius). This difference in behavior is likely a result of the shape of the condensate boundary being directly impacted by the value of the smoothing parameter. However, as discussed in the main text, the smoothing parameter should typically be kept close to 1.0 and should only be increased when microscope noise cannot be reduced using other methods.

#### Bayesian optimisation tool for image parameter estimation

Although we have shown that the analysis performed by *FlickerPrint* is robust to changes in imaging parameters, it is still important to ensure that optimal parameters are selected. A good set of imaging parameters result in a high number of valid objects of interest (objects which pass all filtering steps) and a low number of rejected objects (those which do not pass one of the filters for a given frame). To help with choice of parameters, *FlickerPrint* contains an optimisation tool to determine the optimal imaging parameters (minimum object intensity, and maximum and minimum object radius) to maximise the number of condensates found in an assay.

In order to do so, it is necessary to quantify how good a given set of parameters is. For this, two objectives are used: i) the number of usable condensates found need to be as high as possible and ii) the number of unusable condensates should be as low as possible. Typically, these two objectives do not align with each other; if the number of usable condensates is high, then the number of unusable condensates is often also high. Conversely, if the number of unusable condensates is low, the number of usable condensates is typically also low. It is therefore necessary to balance these two objectives against each other.

As a given assay often contains multiple videos, each with hundreds of frames, repetitive evaluation of every frame in every video would be very computationally expensive. Therefore, only a sample set of frames are used for the parameter estimation. The selected frames are taken from a subset, *E*_sub_, of up to 12 videos, sampled from a uniform distribution of all the provided videos. For each video 2 random frames, *I*_sub_ are analyzed, again sampled from a uniform distribution.

If *N* is the number of videos in *E*_sub_, *M* is the number of frames in *I*_sub_ and *θ* is a set of imaging parameters parameters, then the two objectives can be written as

(Equation 7) $$F_{1}\left(\theta ,E_{\text{sub}}\right)=\sum_{I_{\text{sub}}\in E_{\text{sub}}}\min_{f\in I_{\text{sub}}}{\text{DoG}}_{\text{usable}}\left(\theta ,f\right)\text{,}$$

and

(Equation 8) $$F_{2}\left(\theta ,E_{\text{sub}}\right)=-\frac{1}{N}\sum_{I_{\text{sub}}\in E_{\text{sub}}}\left(\frac{1}{M}\sum_{f\in I_{\text{sub}}}{\text{DoG}}_{\text{unusable}}\left(\theta ,f\right)\right)\text{,}$$

where DoG_usable_ and DoG_unusable_ are instances of the Difference of Gaussians algorithm that return the number of usable and unusable objects in frame *f*, respectively.

$F_{1}$ corresponds to objective i) and tries to maximize the sum of the number of usable objects of interest for the frame with the fewest number of usable objects in each image. This approach is chosen to find a parameter set where the floor of found condensates is increased as much as possible. This has the benefit that objects are present in as many images as possible, allowing for better analysis with *FlickerPrint*.

$F_{2}$ corresponds to objective ii) and tries to minimize the mean number of unusable objects of interest over all images, where each image is weighted by the mean number of unusable objects found in its frames. Contrary to objective i), it is not necessary to worry about continuous existence of the objects, only the total number. The goal is therefore to try to find a parameter set that reduces the mean number of unusable objects of interest.

Since *FlickerPrint* performs best with a high number of usable condensates, objective i) is prioritized over objective ii). Using this, the problem can be described as a multi-objective optimisation problem with ranked objectives.^57^ The problem can be written as a lexicographic optimisation

(Equation 9) $$\text{lex}\;\text{max}\;\;\;F_{1}(\theta ;E_{\text{sub}})F_{2}(\theta ,E_{\text{sub}})$$

(Equation 9a) s.t.θ∈Θ,

where Θ is the set of all possible imaging parameters. Equation 9 is solved with a sequential algorithm, meaning that the problem is divided into two single-objective optimisation problems which are solved sequentially.

The first problem is given by

(Equation 10) $$n_{\max}=\max\;F_{1}\left(\theta ;E_{\text{sub}}\right)$$

(Equation 10a) s.t.θ∈Θ

with *n*_*max*_ being the maximum number of usable condensates found in *E*_sub_.

The second optimisation problem is given by

(Equation 11) $$\max\;F_{2}\left(\theta ;E_{\text{sub}}\right)$$

(Equation 11a) s.t.θ∈Θ,

(Equation 11b) $$F_{1}\left(\theta ;E_{\text{sub}}\right)\geq 0.9n_{\max}\text{.}$$

The second optimisation is constrained by the maximum number of usable condensates found in the first, higher prioritized optimization. While the ideal choice of the constraint is dependent on the assay, we found that 0.9 resulted in good results in most tested cases.

Since the evaluation of $F_{1}$ and $F_{2}$ is of high cost and since DoG_usable_ and DoG_unusable_ do not take on a functional form, Bayesian optimization is used to solve Equation 9.^45^^,^^58^

Figure S7 demonstrates an application of the Bayesian optimisation tool to stress granules induced in U2OS cells; Figure S7 A shows that the optimisation tool is able to successfully converge on a set of parameters *θ* to minimise the objective function. The variation seen in the start and endpoints of the optimisation are due to different frames being selected for each repeat. However, as demonstrated in Figure S7B, these differences lead to minimal variation in the optimised parameters. Taken together with the parameter robustness analysis, the optimisation tool yields stable distributions of the mechanical properties of the objects of interest at the population level.

### Quantification and statistical analysis

#### Population filtering

In addition to the requirements placed on objects of interest (that objects can be located using the provided imaging parameters and that their boundary can be described by a continuous radial function) which act on a frame-by-frame and object-by-object basis, filtering can be applied at the population level. In this work, the following filters were applied to all population-level analyses: interfacial tension *σ* > 10^−10^N/m, continuous boundary in >60% of frames, fitting error *ε* < 0.5 and |*ε*[*F*(*σ*,*κ*)]-*ε*[*F*(*σ*)]|>0.03, where *ε*[*F*(*σ*,*κ*)]-*ε*[*F*(*σ*)] is the difference in fitting error between models which include contributions from both interfacial tension and bending rigidity or interfacial tension only.

#### Statistical quantities

All analysis conducted using *FlickerPrint* is initially output as raw data. For distributions which follow a log-normal distribution such as those for interfacial tension and bending rigidity, statistical values are reported as the geometric mean and the geometric mean ±1 geometric standard deviation (this is equivalent to the 68% confidence interval for a normal distribution). For all other distributions, statistical values are reported as the mean and the mean ±1 standard deviation.

### Additional resources

The FlickerPrint package is available through the PyPI, the Python package manager and on GitHub.^47^^,^^59^

## Supplemental information

Document S1. Figures S1–S7 and Tables S1 and S2

Document S2. Article plus supplemental information
