# Supplementary material for: Non-invasive measurement of biomolecular condensate interfacial tension and bending rigidity
Source: Cell Rep Methods. 2025 Nov 11;5(12):101223. doi: 10.1016/j.crmeth.2025.101223 (PMC12859508; doi:10.1016/j.crmeth.2025.101223)
Supplement: Document S1. Figures S1–S7 and Tables S1 and S2 [file mmc1.pdf]

Cell Reports Methods, Volume *5*

Supplemental information

Non-invasive measurement of biomolecular condensate interfacial tension and bending rigidity

Thomas A. Williamson, Jack O. Law, Thomas Stevenson, Fynn Wolf, Carl M. Jones, Endre S. Tønnessen, Sushma N. Grellscheid, and Halim Kusumaatmaja

Supplementary Figures

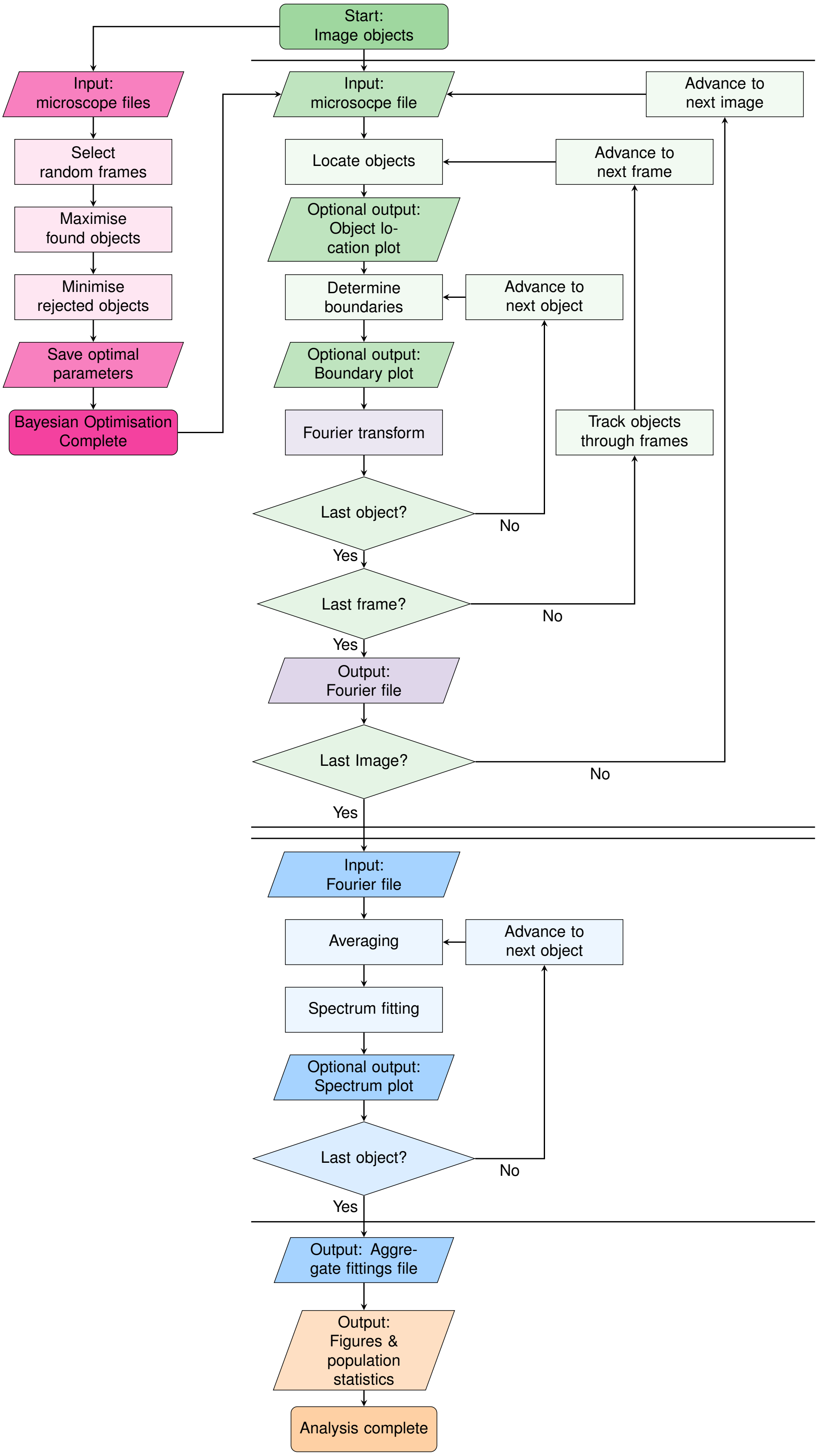

Figure S1: **A flowchart detailing the full workflow of *FlickerPrint*. Related to Figure 1.** The workflow is split into five stages; location of granules in a microscope image (green), Fourier Transform of the boundary fluctuations (purple), fitting of the theoretical power spectrum (blue), Bayesian parameter estimation (pink) and analysis of population-level statistics (orange). Steps in between parallel lines indicate that work on multiple microscope images can be parallelised across multiple cores.

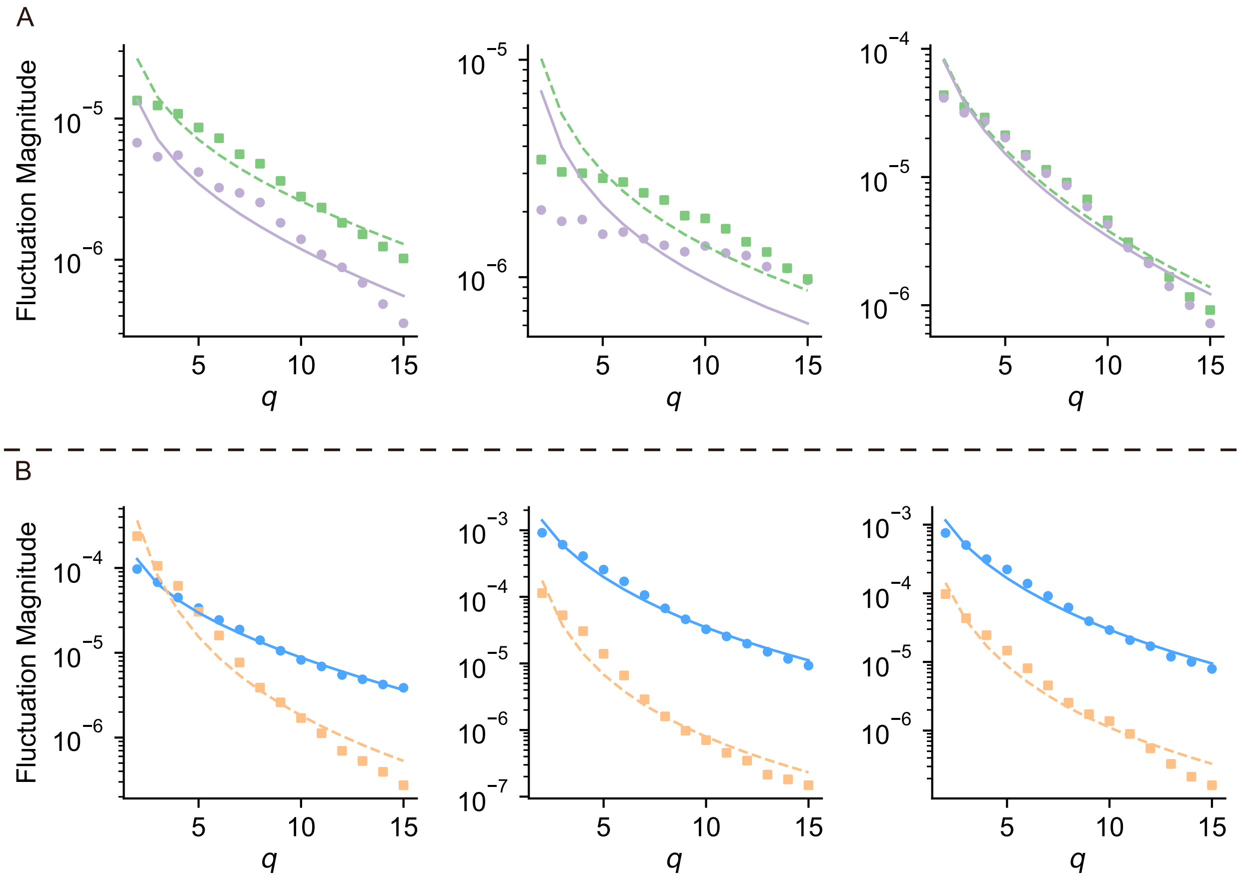

Figure S2: **Wetting and imaging plane position can have a significant impact on the fluctuation spectrum of the objects of interest. Related to Figure 3.** (**A**) Example spectra from the *in-vitro* condensates shown in Figure 3, where the condensates have wetted onto the surface of the vessel. For each condensate (different column), the spectra are shown for a lower (purple circles) and higher (green squares) imaging plane. These spectra curve in the opposite direction to the prediction from equation 1 of the main text. Therefore, condensates which wet onto other objects should be avoided. (**B**) Example spectra for *in-vitro* condensates without substantial wetting, where each condensate is imaged in two planes. Imaging in a plane away from the equator (orange squares) causes the spectrum to change shape and often leads to reduced fluctuation amplitude; imaging close to the equator (blue circles) allows for a better fit to equation 1 of the main text. Note the difference in y-scale, compared with Figure 3 E.

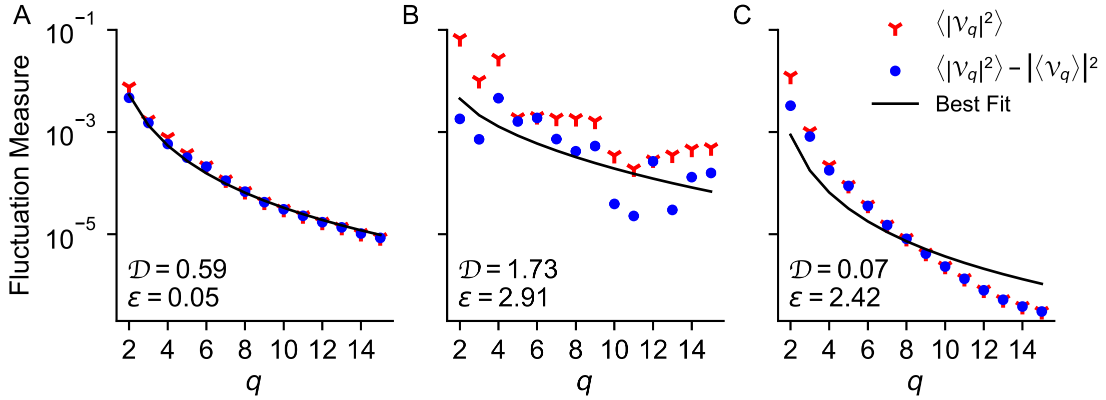

Figure S3: **The fitting error ε and Durbin-Watson statistic $\mathcal{D}$ can be used as a measure of the goodness of fit of the analytic power spectrum to the measured fluctuation amplitudes [S1]. Related to Figure 4.** Three example spectra of stress granules in U2OS cells are shown. In (**A**), the model is a good fit to the experimental spectrum; in (**B**), the experimental spectrum is very noisy and in (**C**), the model does not fit the experimental spectrum well.

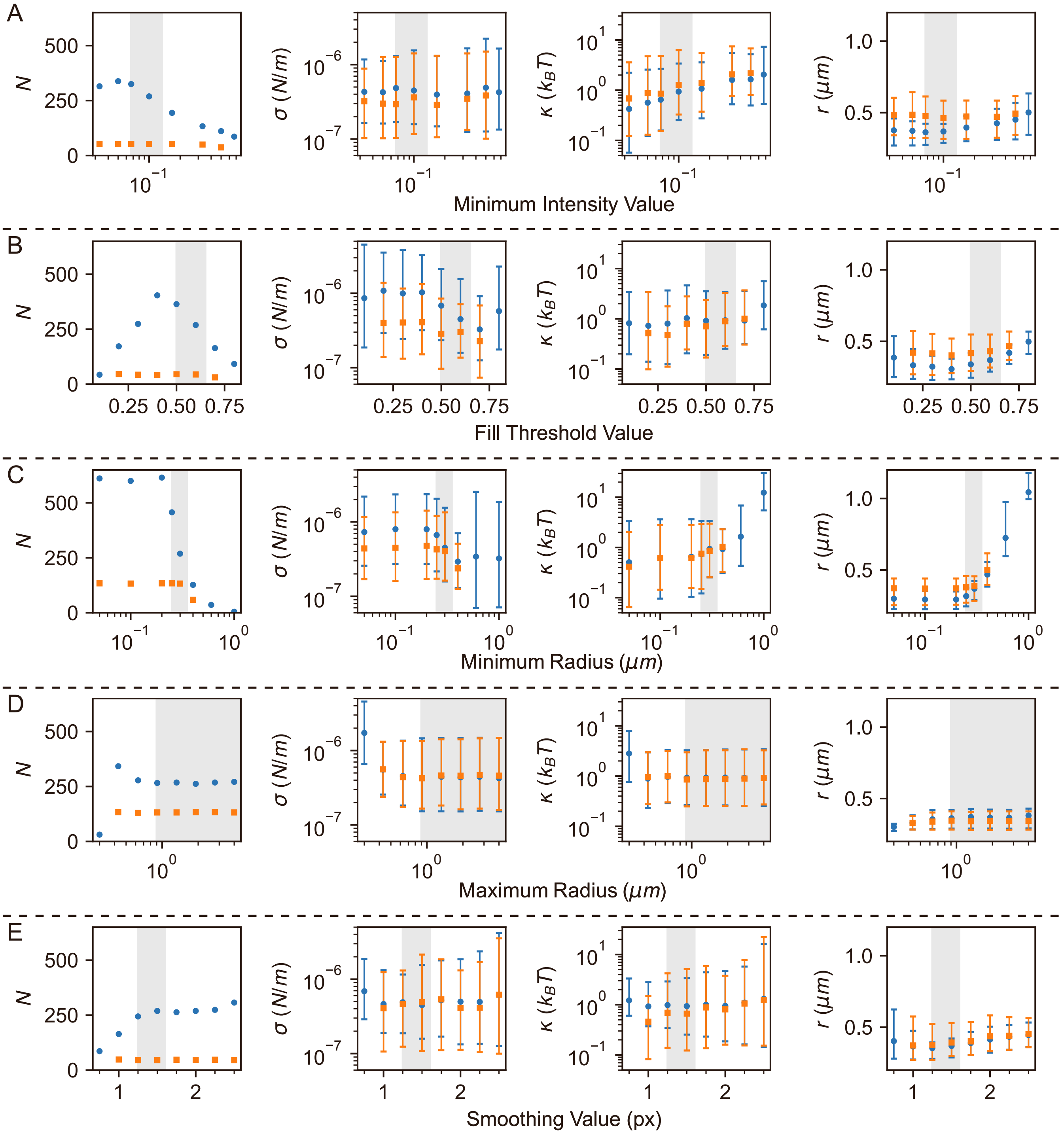

Figure S4: **The parameter distributions output by *FlickerPrint* are robust to variations in the imaging parameters used to configure the analysis. Related to Figure 5 and STAR Methods.** The values for minimum object intensity (**A**), flood fill threshold (**B**), minimum object radius (**C**), maximum radius (**D**) and the smoothing parameter (**E**) are varied independently for analysis of one video of stress granules in U2OS cells. The effect on the number of condensates which are found and pass the filtering stages N, the interfacial tension σ, bending rigidity κ and mean condensate radius r are plotted. Points show the mean value of the output distributions; error bars show $\pm$ 1 standard deviation. Blue circles show data for all condensates which pass the filtering steps; orange squares show distributions which only contain condensates which are consistently found across 75% of analyses of the same parameter. Where less than 100 condensates from one dataset passed the filters, tracking between datasets was not performed. Light grey regions indicate the window of optimal parameters for the image analysed.

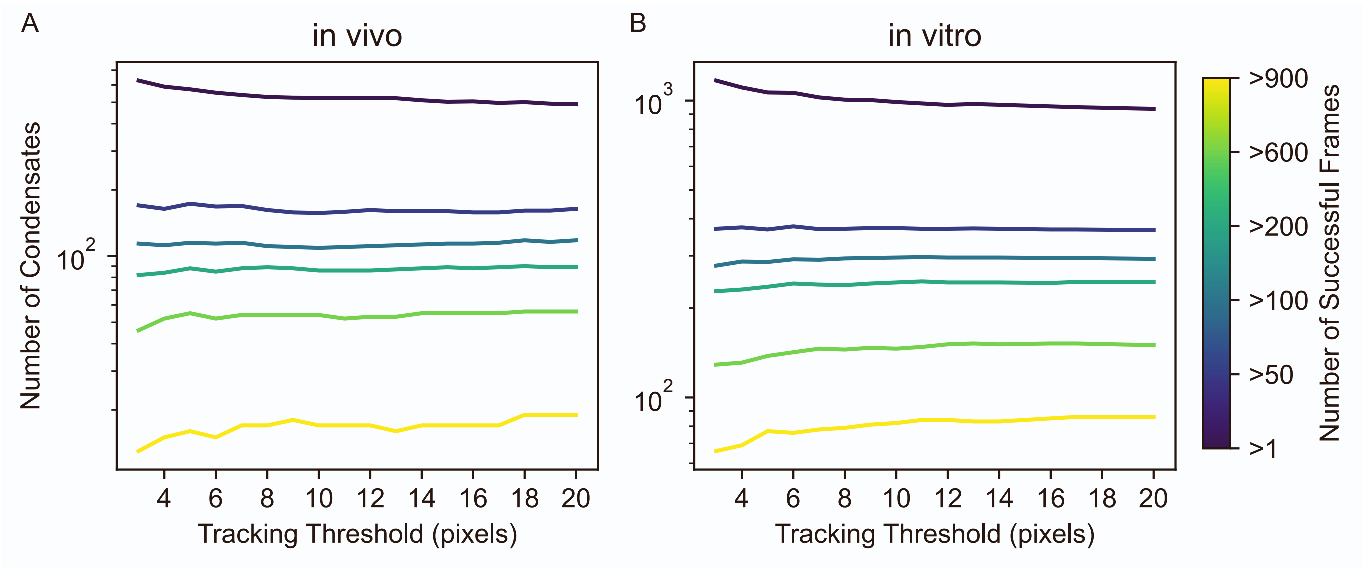

Figure S5: **The threshold for tracking condensates between frames can be adjusted within *FlickerPrint*. Related to STAR Methods.** Typically, there is little impact on the identified condensates when the threshold is increased above 15 pixels. Analysis was conducted on the systems shown in Figure 2 (A,B) of the main text.

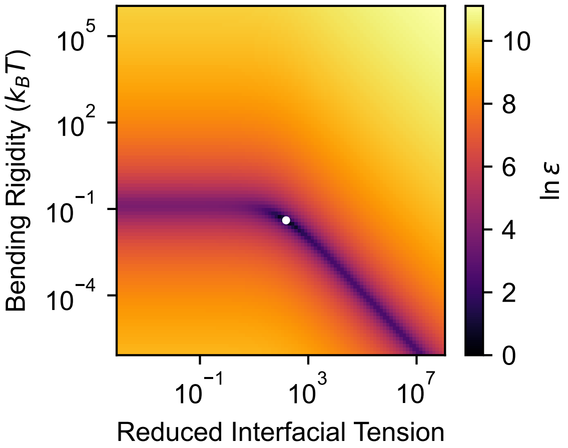

Figure S6: **A heat-map showing the error surface $\varepsilon(\bar{\sigma}, \kappa)$ for a representative stress granule. Related to STAR Methods.** The minimum of the surface is indicated with a white dot. The x-axis shows reduced interfacial tension $\bar{\sigma} = \frac{\sigma R^2}{\kappa}$.

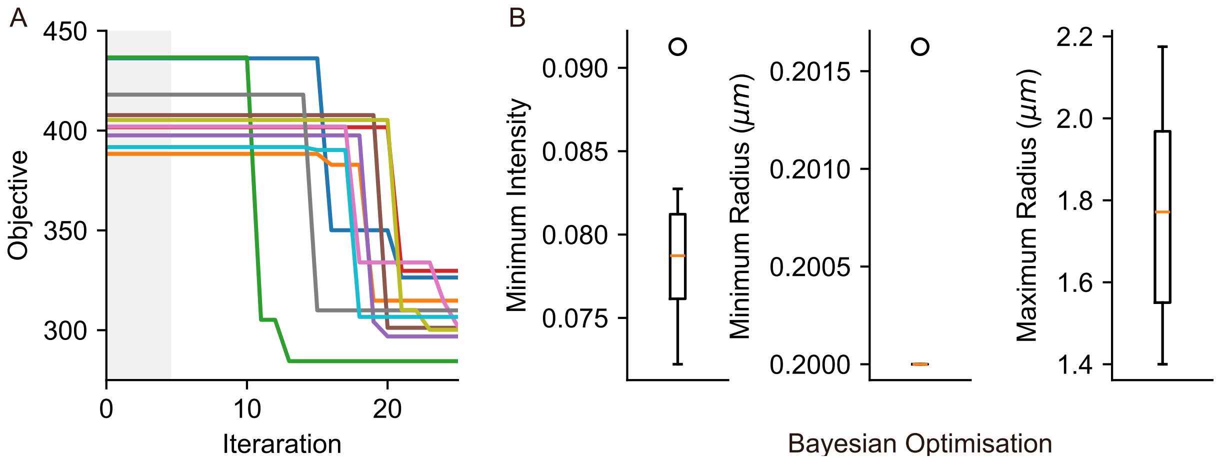

Figure S7: **Bayesian Optimisation can be used to determine appropriate imaging parameters for *FlickerPrint* analysis. Related to STAR Methods.** (**A**) Value of the objective function given by equation S7 with iteration count for the lexicographic optimisation, performed on images of stress granules in U2OS cells. 10 repeats, each using 5 frames from 3 randomly selected images are shown. The shaded grey region is the ‘burn in’ period (5 iterations) for the optimisation. (**B**) Box plots showing the spread of optimised imaging parameters (minimum object intensity, minimum radius and maximum radius), as determined by the optimisations performed in (A).

Supplementary Tables

Frame Rate (fps)	Number of Condensates	Interfacial Tension $^{+1GSD}_{-1GSD}$ $(\mu N/m)$	Bending Rigidity $^{+1GSD}_{-1GSD}$ (k_BT)	Mean Radius $^{+1SD}_{-1SD}$ (μm)
6	121	$0.259^{+0.326}_{-0.135}$	$0.92^{+1.44}_{-0.53}$	$0.40^{+0.08}_{-0.09}$
8	121	$0.281^{+0.394}_{-0.161}$	$0.92^{+1.59}_{-0.56}$	$0.40^{+0.08}_{-0.10}$
12	121	$0.297^{+0.331}_{-0.160}$	$0.94^{+1.32}_{-0.55}$	$0.40^{+0.08}_{-0.10}$
24	121	$0.302^{+0.428}_{-0.171}$	$0.91^{+1.38}_{-0.51}$	$0.40^{+0.08}_{-0.10}$

Table S1: **Statistics for parameter distributions of the same two videos of stress granules in U2OS cells, analysed at differing effective frame rates. Related to Figure 5.** Only condensates which are found, analysed and pass the filtering steps in all four analyses are included in the statistics. Consequently, there is very little deviation in the parameter distributions. Interfacial tension and bending rigidity distributions are shown in Figure 5 A, B of the main text.

Frame Rate (fps)	Number of Condensates	Interfacial Tension $^{+1GSD}_{-1GSD}$ $(\mu N/m)$	Bending Rigidity $^{+1GSD}_{-1GSD}$ (k_BT)	Mean Radius $^{+1SD}_{-1SD}$ (μm)
6	177	$0.299^{+0.581}_{-0.173}$	$0.93^{+1.83}_{-0.0.63}$	$0.38^{+0.05}_{-0.08}$
8	227	$0.316^{+0.442}_{-0.179}$	$0.88^{+1.76}_{-0.56}$	$0.36^{+0.04}_{-0.07}$
12	268	$0.367^{+0.679}_{-0.235}$	$1.06^{+2.37}_{-0.72}$	$0.36^{+0.05}_{-0.07}$
24	400	$0.417^{+0.646}_{-0.253}$	$1.07^{+2.07}_{-0.72}$	$0.34^{+0.03}_{-0.06}$

Table S2: **Parameter distribution statistics for two videos of stress granules in U2OS cells, analysed at differing effective frame rates. Related to Figure 5.** Similar to Table S1, but statistics are for all condensates found in each set of analysis, regardless of whether they were found in other analyses. More condensates can be tracked successfully and are visible in sufficiently many frames for their fluctuations to be averaged at higher frame rates. This leads to variations in the parameter distributions.

References

[S1] Durbin, J., and Watson, G. S. (1950). Testing for serial correlation in least squares regression: I. Biometrika 37, 409–428. doi:10.2307/2332391.
